# Monkey fossils do not negate cosmogenic dating at Sterkfontein

**DOI:** 10.1073/pnas.2300314120

**Published:** 2023-03-20

**Authors:** Darryl E. Granger, Dominic Stratford, Laurent Bruxelles, Jason L. Heaton, Travis Rayne Pickering, Kathleen Kuman, Ronald J. Clarke

**Affiliations:** ^a^Department of Earth, Atmospheric, and Planetary Sciences, Purdue University, West Lafayette, IN 47907; ^b^School of Geography, Archaeology and Environmental Studies, University of the Witwatersrand, Johannesburg WITS 2050, South Africa; ^c^Travaux et Recherches Archéologiques sur les Cultures, les Espaces et les Sociétés, UMR 5608 of the CNRS, Jean Jaurès University, Toulouse 31058, France; ^d^Department of Biology, Birmingham-Southern College, Birmingham, AL 35254; ^e^Department of Anthropology, University of Wisconsin, Madison, WI 53706; ^f^Evolutionary Studies Institute, University of the Witwatersrand, Johannesburg WITS 2050, South Africa

Frost et al. (1) show that molars of the East African *Theropithecus oswaldi* lineage become systematically larger from 4.0 to 0.5 My. They use this trend to infer ages for various South African fossil sites, assuming no clinal variation in tooth size over the continent. They estimate an age of ca. 2.4 My from the large *T. oswaldi darti* teeth at Makapansgat. Sterkfontein Members 4 and 2 lack *Theropithecus* but preserve other cercopithecid species similar to Makapansgat, so they propose a similar age, rejecting radiometric dates and stratigraphic observations (2) placing Sterkfontein Members 4 and 2 from ca. 3.4 to 3.7 My. We do not question that tooth size can be helpful for relative dating in East Africa but rather challenge the extrapolation of inferred ages to Sterkfontein. Frost et al. have based their age estimate for Sterkfontein mainly on paleomagnetism and U–Pb dating of flowstones and the presence of *Cercopithecoides williamsi*, “true” *Papio*, and *Parapapio*, which they compare with Makapansgat. These lines of argument are problematic:

1. Paleomagnetism and U–Pb measurements at Sterkfontein derive exclusively from flowstones that are intrusive or out of stratigraphic context and are, therefore, younger than the fossil-bearing breccia (2–4).
2. *C. williamsi* shows substantial variation across sites and may not be conspecific with fossils in East Africa (5). Sterkfontein *Cercopithecoides*, and also *Parapapio*, could be older than Makapansgat.
3. Heaton (6) demonstrated that “true” *Papio* (<2.3 My) was misidentified in Member 4: SWP 31 is in fact **Papio* izodi*; moreover, it did not derive from “more recent controlled excavations,” as claimed but from blasting operations, as did other younger specimens in early collections.

We, therefore, disagree that Sterkfontein must be <3 My, which would require that radiometric dating of breccias (2, 7) is flawed. Previous criticisms (8) suggesting that older cave sediments could be mixed with younger fossils do not apply (2). Our dating and stratigraphy (2) show that Member 4 sediments are intact and that the historic faunal assemblage is mixed because some younger fossils from overlying Member 5 (not recognized at the time) were collected from blasted breccias and assumed to belong to Member 4. In limited areas affected by deep solution pockets, younger fossils were also mistakenly assigned to Member 4 because no record was kept of these sediments. In addition, significant differences in *Australopithecus* and *Chasmaporthetes* fossils are consistent with Member 2 being paleontologically older than Member 4 (2, 9, 10).

It is logical and parsimonious that historic problems caused by blasting operations and the lack of stratigraphic detail prior to the 1990s caused such confusion. The absence of *Theropithecus* in the cercopithecid-rich Members 4 and 2, along with its minimal presence in Member 5, highlights differences between Sterkfontein and Makapansgat (where *Theropithecus* is common), which cannot be ignored. *Theropithecus* tooth size in South Africa may be biased by clinal or regional variation. Given these uncertainties, the absence of *Theropithecus*, and evidence for a mixed assemblage, the faunal evidence does not warrant overturning robust, repeated, and internally consistent radiometric dating of the Sterkfontein breccia.
